# Supplementary material for: SATB2 induction of a neural crest mesenchyme-like program drives melanoma invasion and drug resistance
Source: eLife. 2021 Feb 2;10:e64370. doi: 10.7554/eLife.64370 (PMC7880683; doi:10.7554/eLife.64370)
Supplement: Transparent reporting form [file elife-64370-transrepform.docx]

***eLife’s* transparent reporting form**

We encourage authors to provide detailed information *within their submission* to facilitate the interpretation and replication of experiments. Authors can upload supporting documentation to indicate the use of appropriate reporting guidelines for health-related research (see [EQUATOR Network](http://www.equator-network.org/%20)), life science research (see the [BioSharing Information Resource](https://biosharing.org/)), or the [ARRIVE guidelines](http://www.plosbiology.org/article/info:doi/10.1371/journal.pbio.1000412) for reporting work involving animal research. Where applicable, authors should refer to any relevant reporting standards documents in this form.

If you have any questions, please consult our Journal Policies and/or contact us: [editorial@elifesciences.org](mailto:editorial@elifesciences.org).

**Sample-size estimation**

- You should state whether an appropriate sample size was computed when the study was being designed
- You should state the statistical method of sample size computation and any required assumptions
- If no explicit power analysis was used, you should describe how you decided what sample (replicate) size (number) to use

Please outline where this information can be found within the submission (e.g., sections or figure legends), or explain why this information doesn’t apply to your submission:

Sample size for the experiments is specified in the figures, figure legends or methods section. For in primary tumor transgenic the minimum cohort size per injection was 40 animals as previously defined (Ceol et al., 2011). We pre-established criteria to exclude/censor animals if they died without having been examined for tumor formation.

**Replicates**

- You should report how often each experiment was performed
- You should include a definition of biological versus technical replication
- The data obtained should be provided and sufficient information should be provided to indicate the number of independent biological and/or technical replicates
- If you encountered any outliers, you should describe how these were handled
- Criteria for exclusion/inclusion of data should be clearly stated
- High-throughput sequence data should be uploaded before submission, with a private link for reviewers provided (these are available from both GEO and ArrayExpress)

Please outline where this information can be found within the submission (e.g., sections or figure legends), or explain why this information doesn’t apply to your submission:

This information is generally included in the figure legends unless it can be surmised from the figure itself, for example the number of points indicating number of replicates. This would be stated in the legend. N numbers are either stated in the legend or indicated in the graph itself. Screening pool were tested once, while SATB2, EGFP and CCND1 were repeated at least twice. RNA-seq was conducted on 3 biological replicates both for primary tumors (MCR:EGFP and MCR:SATB2) and SKMEL2 iSATB2 cell line. For qPCR on primary tumors there were at least 10 biological replicates run in technical triplicates at least twice. Two biological replicates were run for human cell lines qPCR seeded in technical triplicates, and a biological sample with technical triplicates was run for primary human melanocytes. Gelatin and proliferation assays were seeded in technical triplicates and repeated in 3 independent experiments. For allograft assay between 3-5 donors were used.

**Statistical reporting**

- Statistical analysis methods should be described and justified
- Raw data should be presented in figures whenever informative to do so (typically when N per group is less than 10)
- For each experiment, you should identify the statistical tests used, exact values of N, definitions of center, methods of multiple test correction, and dispersion and precision measures (e.g., mean, median, SD, SEM, confidence intervals; and, for the major substantive results, a measure of effect size (e.g., Pearson's r, Cohen's d)
- Report exact p-values wherever possible alongside the summary statistics and 95% confidence intervals. These should be reported for all key questions and not only when the p-value is less than 0.05.

Please outline where this information can be found within the submission (e.g., sections or figure legends), or explain why this information doesn’t apply to your submission:

This information can be found in the figure legends, methods and dedicated ‘statistics’ method section.

(For large datasets, or papers with a very large number of statistical tests, you may upload a single table file with tests, Ns, etc., with reference to sections in the manuscript.)

**Group allocation**

- Indicate how samples were allocated into experimental groups (in the case of clinical studies, please specify allocation to treatment method); if randomization was used, please also state if restricted randomization was applied
- Indicate if masking was used during group allocation, data collection and/or data analysis

Please outline where this information can be found within the submission (e.g., sections or figure legends), or explain why this information doesn’t apply to your submission:

Experiments to quantify proliferation by PH3 immunohistochemistry, and cells with degraded 488-conjungated gelatin were scored blindly. Irradiated casper zebrafish were randomized between transplantation groups. No randomization or blinding was used for MCR tumor models or allografts.

**Additional data files (“source data”)**

- We encourage you to upload relevant additional data files, such as numerical data that are represented as a graph in a figure, or as a summary table
- Where provided, these should be in the most useful format, and they can be uploaded as “Source data” files linked to a main figure or table
- Include model definition files including the full list of parameters used
- Include code used for data analysis (e.g., R, MatLab)
- Avoid stating that data files are “available upon request”

Please indicate the figures or tables for which source data files have been provided:

Excel source file was provided for the following figures:

Figure 1

Figure 1 –Supplement 1
